# DREADDs‐Based Chemogenetics Induced Slow Transit Constipation via Inhibition of Enteric Neurons

**DOI:** 10.1111/1751-2980.13344

**Published:** 2025-04-14

**Authors:** Xin Yi Lu, Yu Xiang Wen, Ni Jiang, Si Qi Zhou, Tian Yang, Liang Liang Shi, Hui Min Guo, Wei Zhang, Qi Peng Zhang, Ni Na Zhang

**Affiliations:** ^1^ Department of Gastroenterology Nanjing Drum Tower Hospital Clinical College of Nanjing University of Chinese Medicine Nanjing Jiangsu Province China; ^2^ Department of Gastroenterology Nanjing University Medical School Affiliated Nanjing Drum Tower Hospital Nanjing Jiangsu Province China; ^3^ State Key Laboratory of Pharmaceutical Biotechnology, Institute for Brain Sciences, School of Life Sciences Nanjing University Nanjing Jiangsu Province China; ^4^ Nanjing Drum Tower Hospital Clinical College of Nanjing Medical University Nanjing Jiangsu Province China

**Keywords:** calcium transient, designer receptors exclusively activated by designer drugs, enteric nervous system, gastrointestinal motility

## Abstract

**Objectives:**

Designer receptors exclusively activated by designer drugs (DREADDs)‐based chemogenetic tools are commonly used to activate or silence targeted neurons by the agonistic ligand deschloroclozapine (DCZ). This study aimed to establish a Gi‐DREADD‐based murine model of slow transit constipation (STC) and elucidate its pathophysiological mechanisms.

**Methods:**

Adeno‐associated virus (AAV) 9‐hM4Di was injected into the intestinal wall of mice, and colonic motility was evaluated. The efficiency and immunogenicity of AAV9‐hM4Di transduction in the enteric nervous system (ENS) were evaluated. Nitric oxide (NO), acetylcholine (ACh), and substance P (SP) in the colonic tissues and serum samples were analyzed. Calcium (Ca^2+^) imaging was performed to evaluate the responses of AAV9‐hM4Di on enteric nerves.

**Results:**

AAV9‐hM4Di‐treated mice showed gastrointestinal motility dysfunction, including reduced fecal pellets and decreased fecal mass and water content. Electrophysiological recording of muscle contraction in the isolated colonic tissues from the chemogenetic mice showed decreased frequency and amplitude after DCZ treatment. The mice treated with AAV9‐hM4Di showed the highest levels of transduction in the myenteric plexuses of the ENS. There were no differences in transduction in neuronal nitric oxide synthase (nNOS) and choline acetyltransferase (ChAT) neurons. Gi‐DREADDs significantly downregulated ACh but not NO or SP expression in the distal colon in the chemogenetic mice. Ca^2+^ transient in neurons of ENS in chemogenetic mice was strongly inhibited by DCZ.

**Conclusions:**

It is feasible to apply the DREADDs‐based chemogenetic tools to the ENS. Gi‐DREADDs can selectively modulate the ENS, inducing STC without excitatory‐neural bias, offering targeted neuromodulation for gastrointestinal motility disorders.

## Graphical abstract

Designer receptors exclusively activated by designer drugs (DREADDs)‐based chemogenetic tools were used to induce murine model of slow transit constipation. Adeno‐associated virus (AAV) 9‐hM4Di or normal saline (control) was injected into six sites spread out over the whole colon of mice, and the indicators of colonic motility were evaluated after deschloroclozapine (DCZ) injection. Gi‐DREADDs can selectively modulate the enteric nervous system (ENS) to induce constipation and provide targeted neuromodulation for gastrointestinal motility disorders.

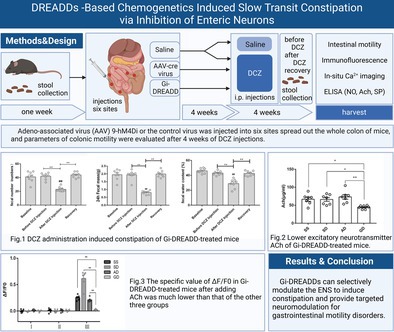

## 1 Introduction

Slow transit constipation (STC) is one of the most prevalent gastrointestinal (GI) diseases characterized by a prolonged colonic transit with symptoms including difficulty in and reduced frequency of defecation, dry and hard stool, accompanied by abdominal distension and pain [1]. Compared with healthy individuals, the quality of life (QoL) of patients with constipation has been reported to be more impaired [2]. However, so far, the mechanisms of STC remain to be investigated and the treatment strategies are still limited.

The enteric nervous system (ENS), relatively independent of the central nervous system (CNS), regulates peristalsis of the GI tract [3]. Enteric excitatory and inhibitory muscle motor neurons mediate the SIP syncytium, which is composed of smooth muscle cells (SMCs), interstitial cells of Cajal (ICC), and platelet‐derived growth factor receptor‐α‐positive (PDGFRα^+^) cells, to generate colonic motility. Dysfunction of the ENS disrupts GI motility, which is the main characteristic of STC. Several abnormal motor symptoms have been described in STC, including changes in rectosigmoid activity [4], impaired colonic propulsive function [5], and decreased electrical activity of the colon [6]. The motor neurons release the corresponding neurotransmitters, including nitric oxide (NO) and acetylcholine (ACh), to act on both ICC and SMCs. Imbalanced expressions of neurotransmitters lead to the dysfunction of distal inhibitory neurons and proximal excitatory neurons, resulting in slow peristaltic movement [7, 8].

Chemogenetic technologies have been shown to enhance or inhibit neuronal activity. Designer receptors exclusively activated by designer drugs (DREADDs), one of the chemogenetic G protein‐coupled receptor (GPCR) signaling platforms, can promote physiologically relevant and reversible patterns of neuronal modulation via interaction with ligands that are not recognized by endogenous receptors, such as deschloroclozapine (DCZ) [9, 10]. Currently, the main DREADD is composed of hM3Dq and hM4Di, both of which originate from the human muscarinic receptor and κ‐opioid receptor (KOR) [11]. DREADD has been reported to be applied for neuronal control in breathing, feeding, and emotional behaviors [12, 13]. McClain et al. [14] found that glial calcium (Ca^2+^) signaling regulated gut motility in a transgenic chemogenetic *GFAP::hM3Dq* mouse model. To date, little is known about the transduction of DREADD receptors to induce abnormalities of the ENS that cause slow transit of the colon. Loperamide has been used to induce STC as it can cause decreased intestinal nerve activity [15, 16, 17]. However, the utility of loperamide is time‐consuming and unstable due to a large individual difference. Moreover, it is not completely conformed to the pathogenesis of decreased activity of the ENS [18, 19].

In the current study, we aimed to evaluate the ability of adeno‐associated virus (AAV)–hM4Di in the induction of STC mouse model, and to investigate the involved mechanisms, trying to provide insights for the utility of DREADD technology in STC and other GI motility disorders in the future.

## 2 Materials and Methods

### 2.1 Animals and Viruses

Male C57BL6/J mice (specific pathogen‐free [SPF] grade, 8–10 weeks old, body weight 25–30 g) were obtained from the Beijing Vital River Laboratory Animal Technology Co. Ltd. (Beijing, China). The mice were housed individually (one mouse in each cage) in a ventilated house at room temperature with a humidity of 50%–70% under the 12‐h light/dark cycle. All mice had a free access to the standard chow and water. All the animal experiments were approved by the Ethics Committee and Animal Welfare Committee of Nanjing Drum Tower Hospital, The Affiliated Hospital of Nanjing University Medical School (Nanjing, Jiangsu Province, China) (no. 2021AE01075). The pAAV9‐CAG‐EGFP‐P2A‐Cre‐WPRE (3.8 × 10^13^ viral genomes per milliliter [VG/mL]) was purchased from OBiO Tech (CN396; Shanghai, China), while pAAV9‐EF1a‐DIO‐hM4D(Gi)‐mCherry (4.45 × 10^13^ VG/mL) was purchased from WZ Biosciences Inc. (pAV202014; Jinan, Shandong Province, China).

### 2.2 Study Design

**FIGURE 1:**
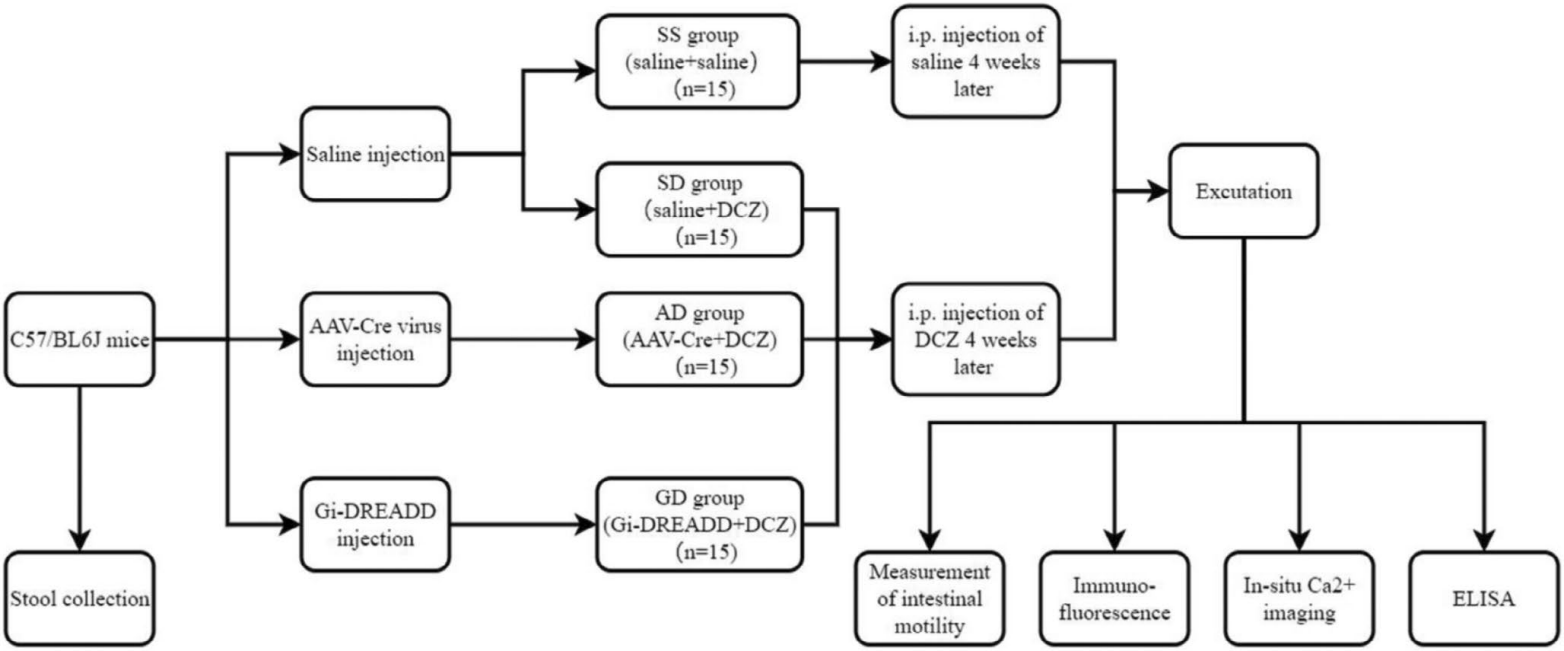
Flowchart of grouping of the mice and the experiment. AAV, adeno‐associated virus; DCZ, deschloroclozapine; i.p., intraperitoneal.

After 1‐week acclimatization and daily stool collection, the mice were randomly divided into the following groups using a random number generator: (a) normal saline + normal saline (SS; *n* = 15); (b) normal saline + DCZ (SD; *n* = 15); (c) AAV‐Cre (pAAV9‐CAG‐EGFP‐P2A‐Cre‐WPRE) + DCZ (AD; *n* = 15); and (d) Gi‐DREADD (pAAV9‐CAG‐EGFP‐P2A‐Cre‐WPRE and pAAV9‐EF1a‐DIO‐hM4Di‐mCherry) + DCZ (GD; *n* = 15). Normal saline or the viruses were injected into the intestinal wall of the mice according to their grouping. Four weeks after, the mice were then intraperitoneally injected with DCZ (HY‐42110; 100 μg/kg body weight; MedChemExpress, Nanjing, Jiangsu Province, China) or normal saline for 7 days. Fecal pellets were collected at baseline, before DCZ injection, after DCZ injection, and at recovery, respectively, each continued for 7 days. The GI transit experiment and colon bead expulsion test were conducted during these four time periods as well. Six mice were randomly selected from each group and sacrificed after DCZ or normal saline injection; blood samples were collected from the orbital sinus, and colonic tissues from the ileocecum to the terminal of the descending colon were harvested for further analysis. The study flowchart and grouping of the mice are shown in Figure 1.

### 2.3 Surgery and injection of the viruses

All mice were anesthetized with 1.5%–3.0% isoflurane. After depilation and sterilization with 75% ethanol, an incision was made in the middle of the abdomen to expose the peritoneal cavity and the entire colon. The pAAV9‐CAG‐EGFP‐P2A‐Cre‐WPRE, with or without pAAV9‐EF1a‐DIO‐hM4D(Gi)‐mCherry, or normal saline, was injected into the wall of the colon according to the grouping of the mice, with 2 μL for each injection and six injections at different sites along the longitudinal axis of the whole colon in each mouse. The injections were made using a 5‐μL syringe (80 016; Hamilton Company, Reno, NV, USA) which was connected to a foot‐operated pump (55–1111; Harvard Apparatus, Holliston, MA, USA), with the tip of the needle locating between the muscular layers during the injection. After each injection, the needle was left for 1 min to prevent reflux. The abdominal incision was then sutured and coated with iodophor. The mice were then placed on an electrothermal pad for recovery at 37°C.

### 2.4 Collection and Examinations of Fecal Samples

To collect fresh fecal samples, all mice were placed individually in the metabolic cages (Shanghai Biowill Co. Ltd., Shanghai, China) and given standard chow and water. The number of fecal samples collected within 24 h was counted and the fecal pellets were weighed immediately to record the wet weight as well as the dry weight (after the fecal samples were dried for 24 h at 38°C in a drying oven [Thermo Fisher Scientific, Waltham, MA, USA]). Fecal water content was then calculated according to the following formula: [(wet weight of the fecal sample − dry weight of the fecal sample)/wet weight of the fecal sample] × 100%. The average values of these variables were calculated during a 7‐day period for further analysis.

### 2.5 Measurement of Intestinal Motility

The collected colonic samples were immersed at 35°C–37°C in Krebs‐Ringer's solution (Beyotime Biotechnology, Shanghai, China), which contained 118.2 mmol/L NaCl, 4.69 mmol/L KCl, 1.17 mmol/L MgSO_4_ • 7H_2_O, 2.52 mmol/L CaCl_2_, 5 mmol/L glucose, 1.39 mmol/L KH_2_PO_4_, 2 mmol/L NaHCO_3_ (pH 7.4), with the addition of different quantities of DCZ (0, 0.1, 1, 10, and 20 nmol/L). One end of the colon was tied to the small hook of the L‐shaped ventilation tube and the other end to the tension transducer. The ventilation tube and intestinal segment were placed in the bath chamber (Yuyan Instruments, Shanghai, China). The tension value was transmitted and collected by the FT‐102 N Muscle Tension Sensor and transferred into the specific waves by using the BL‐420 N Data Acquisition and Processing System. The recording was performed and analyzed using the HW200S Thermostatic Smooth Muscle Experimental System (all from Chengdu Techman Software Co. Ltd., Chengdu, Sichuan Province, China).

### 2.6 Evaluations of Intestinal Transit Function

The mice received 0.3 mL of 0.5% methylcellulose solution with 6% (*w*/*v*) carmine red (Sigma‐Aldrich, St. Louis, MO, USA) through oral gavage, and had free access to standard chow and water till the presence of red fecal pellets for the first time. Gastrointestinal transit time (GITT) was measured as the time period from oral gavage to the first appearance of red fecal pellets.

Colonic bead expulsion test was performed to assess the expulsion rate of peristaltic waves in the colon. The mice were fasted for 12–14 h before the test and were anesthetized with 1% isoflurane. Glass beads (2 mm in diameter) preheated to 37°C were then inserted till 2.5 cm into the distal colon using a silicone feeding needle (Reward Life Technology, Shenzhen, Guangdong Province, China). The mice were then placed on a white sheet, and the time for bead expulsion was recorded in minutes.

### 2.7 Immunohistochemistry

After being washed with 0.9% normal saline at 4°C, the colonic tissues were fixed with a 4% paraformaldehyde solution for 4 h at room temperature and washed with 0.1 mol/L phosphate‐buffered saline (PBS) for 3–5 times and dehydrated with a 30% sucrose solution at 4°C overnight. The mucosal, submucosal, and circular muscular layers of the colonic tissues were removed under a dissecting microscope (Olympus, Tokyo, Japan). The tissues were then attached to the slides and fixed with 4% paraformaldehyde for 10 min. After being rinsed with PBS thrice and blocked with 2% bovine serum albumin (BSA) (Millipore, Sigma‐Aldrich) in PBS with 0.1% (*v*/*v*) Tween 20 (PBST) (Vazyme Biotechnology, Nanjing, Jiangsu Province, China) for 1 h, the preparations were incubated with antibodies to mCherry (43 590, 1:100; Cell Signaling Technology, Danvers, MA, USA), PGP9.5 (66230‐1‐lg, 1:500; Proteintech, Wuhan, Hubei Province, China), 2‐(4‐Amidinophenyl)‐6‐indolecarbamidine dihydrochloride (DAPI) (C1002, 1:5000; Beyotime Biotechnology), choline acetyl‐transferase (ChAT) (PA5‐18518, 1:500; Invitrogen, Thermo Fisher Scientific), or neuronal nitric oxide synthase (nNOS) (37–2800, 1:200; Invitrogen, Thermo Fisher Scientific) for 24 h at 4°C. The colonic tissues were rinsed again and incubated with the secondary antibodies (A‐11001, 1:1000, Goat Anti‐Mouse immunoglobulin [IgG] [H + L] Cross‐Adsorbed Secondary Antibody; or A‐11055, Donkey Anti‐Goat IgG [H + L] Cross‐Adsorbed Secondary Antibody; both from Invitrogen, Thermo Fisher Scientific) for 1 h at room temperature. All the antibodies, except DAPI, were diluted in a blocking buffer containing 2% BSA, while DAPI was diluted by double distilled H_2_O (ddH_2_O). All the specimens were examined under the fluorescence microscope (Olympus).

### 2.8 Enzyme‐Linked Immunosorbent Assay (ELISA)

After the mice were sacrificed, the fresh colonic tissues were cleaned and immersed in PBS at the rate of 1:9 (for 1 g tissue with 9 mL PBS). The colonic tissues were centrifuged at 13 200 ×*g* for 15 min to obtain tissue supernatant. Blood was collected from the orbital sinus and centrifuged at 13 200 ×*g* for 15 min to obtain serum samples. The tissue supernatant and serum samples were then kept at −80°C until analysis for the level of excitatory (Ach and SP) and inhibitory (NO) neurotransmitters according to the instructions of the manufacturers.

### 2.9 In Situ Ca^2+^ Imaging

The colon was put in the ice‐cold Dulbecco's Modified Eagle Medium (DMEM)/F12 (Gibco, Beijing, China) which contained 3 μmol/L nicardipine hydrochloride (MedChemExpress) and 1 μmol/L scopolamine hydrochloride (MedChemExpress) (hereafter referred as “medium”) for 10 min and cut into several segments. The segments were fixed in Sylgard‐coated Petri dishes (Corning, Shanghai, China) and the mucosa was dissected by fine forceps. The dissected tissues were incubated with Dispase (1 U/mL) and Collagenase Type II (150 U/mL) in the medium for 15 min and immersed in 4 μmol/L Fluo‐4 (MedChemExpress) loading solution for 45 min after being washed with ice‐cold inhibitor‐supplemented DMEM/F12 (3 μmol/L nicardipine, 1 μmol/L scopolamine, 1% BSA, 10 mmol/L HEPES, Ca^2+^/Mg^2+^‐free) thrice. Following incubation in the medium with 200 μmol/L probenecid (MedChemExpress) for 15 min, the preparations were observed under confocal fluorescence microscopy, and the live Ca^2+^ images of the neurons were recorded. Fluo‐4 fluorescence was collected as the baseline value. DCZ (20 nmol/L) was added at the 9th min in the SD, AD, and GD groups, while normal saline of the same volume was added in the SS group. The neuronal response was then recorded. The preparations in the four groups were superfused by adding ACh (100 nmol/L) at the 17th min, and the neuronal response was recorded. The region of interest (ROI) was selected, and the corresponding fluorescence intensity was obtained. Changes in the fluorescence intensity were defined as ΔF/F0, which was calculated using the following formula: (fluorescence intensity of ROI − fluorescence intensity at baseline)/fluorescence intensity at baseline.

### 2.10 Statistical Analysis

All the statistical analyses were performed by using the Graphpad Prism 9 (GraphPad Software Inc., La Jolla, CA, USA). Continuous variables were expressed as mean ± standard error of mean (SEM), whereas categorical variables were expressed as numbers and percentages or frequencies. The two‐way analysis of variance (ANOVA) was used to compare the fecal mass and fecal water content, and the amplitude and frequency of colonic peristalsis among groups or different time points. One‐way ANOVA was used to compare the abovementioned indicators in Gi‐DREADD‐treated mice and Ca^2+^ transient, ELISA results among groups or different time points. The characteristics of transduced neuron cells in the Gi‐DREADD‐treated mice were compared by using the Student's *t*‐test. The images in immunofluorescence and Ca^2+^ transient studies were analyzed using the ImageJ 1.8.0 software (National Institutes of Health, Bethesda, MD, USA). *p* < 0.05 was regarded as statistically significant.

## 3 Results

### 3.1 Gi‐DREADD Induced Constipation in Mice

**FIGURE 2:**
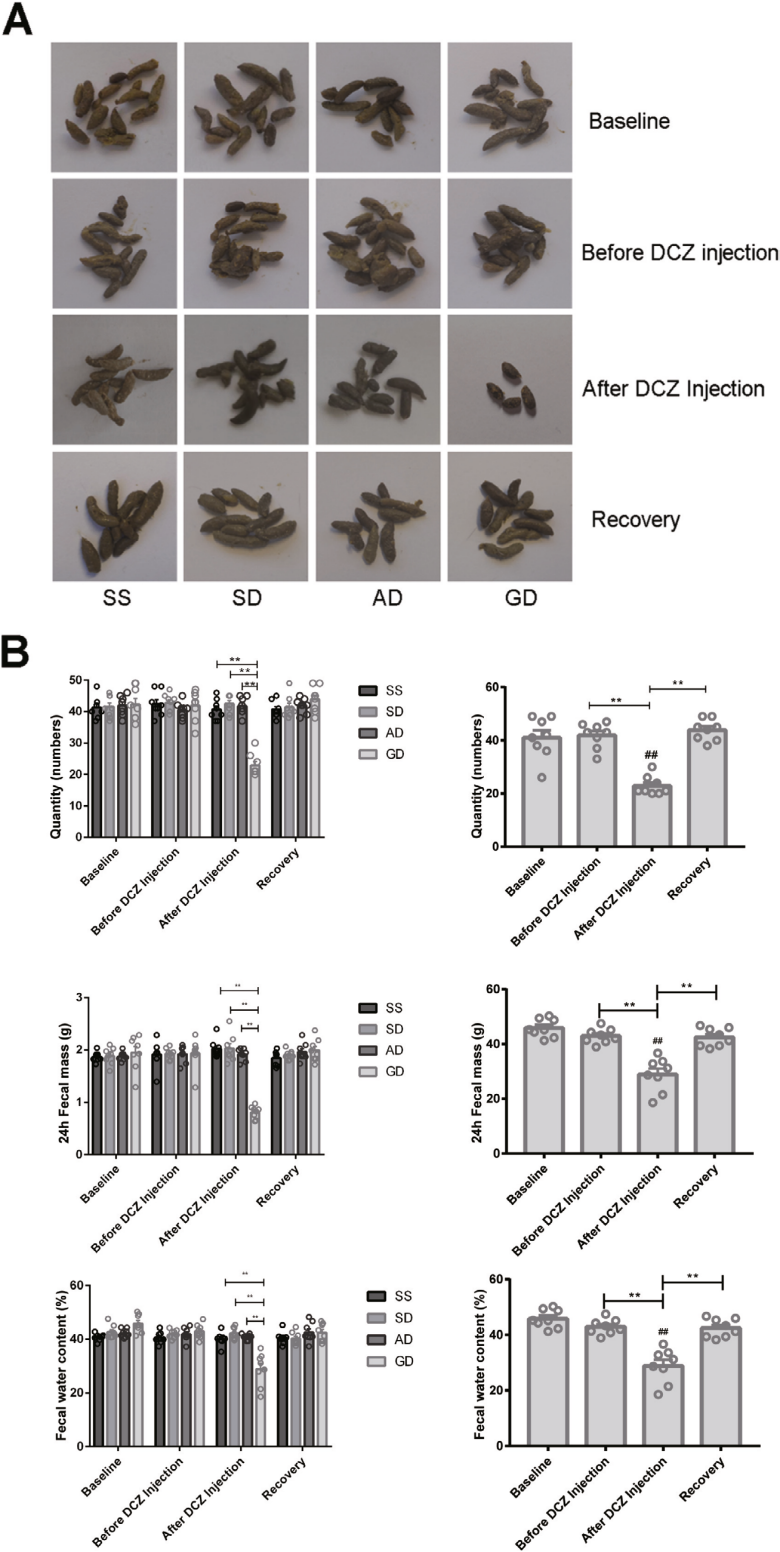
Comparison of fecal quantity, mass, and water content in the normal saline + normal saline (SS), normal saline + deschloroclozapine (DCZ) (SD), adeno‐associated virus (AAV)‐Cre + DCZ (AD), and Gi‐designer receptor exclusively activated by designer drug (Gi‐DREADD) + DCZ (GD) groups. (A) Morphological features of the collected fecal samples of the four groups. (B) Comparisons of 24‐h frequency of defecation, fecal mass, and fecal water content among the four groups (GD group on the right). All data are expressed as mean ± standard error of mean (*n* = 8 per group). *p < 0.05 and **p < 0.01 compared with the GD group after DCZ injection. ^##^ *p* < 0.01 compared with the baseline values of the GD group.

The morphology of the fecal samples of the mice in all groups at baseline, before and after DCZ injection, and at recovery is shown in Figure 2A. After DCZ administration, the fecal quantity, mass, and water content of the Gi‐DREADD‐treated mice were significantly decreased compared with the SS (quantity: 22.88 ± 1.26 vs. 40.63 ± 1.15; mass: 0.81 g ± 0.04 g vs. 2.02 g ± 0.06 g; water content: 28.86% ± 2.18% vs. 40.07% ± 0.86%; all *p* < 0.01), SD (quantity: 22.88 ± 1.26 vs. 42.50 ± 1.04; mass: 0.81 g ± 0.04 g vs. 2.03 g ± 0.09 g; water content: 28.86% ± 2.18% vs. 42.22% ± 0.78%; all *p* < 0.01), and AD groups (quantity: 22.88 ± 1.26 vs. 41.75 ± 1.00; mass: 0.81 g ± 0.04 g vs. 1.92 g ± 0.04 g; water content: 28.86% ± 2.18% vs. 40.89% ± 0.38%; all *p* < 0.01) (Table 1). While fecal quantity, mass, and water content did not differ among groups before DCZ injection or at recovery (Figure 2B). We also observed that, in the GD group, DCZ administration for 1 week decreased the fecal quantity, mass, and water content compared with those before DCZ injection (quantity: 22.88 ± 1.26 vs. 41.00 ± 2.75; mass: 0.81 g ± 0.04 g vs. 1.95 g ± 0.11 g; water content: 28.86% ± 2.18% vs. 45.84% ± 1.17%; all *p* < 0.01). Interestingly, at recovery, all these three indicators of the Gi‐DREADD‐treated mice reverted to those before DCZ injection.

**TABLE 1:** Comparisons of 24‐h fecal quantity, fecal mass, and fecal water content among the saline + saline (SS), saline + deschloroclozapine (DCZ) (SD), adeno‐associated virus (AAV)‐Cre + DCZ (AD), and Gi‐designer receptor exclusively activated by designer drug (Gi‐DREADD) + DCZ (GD) groups.

Groups (*n* = 8 per group)	After DCZ injection	
	Mean ± SEM	*p* value*
Fecal quantity (*n*)		
SS	40.63 ± 1.15	< 0.01
SD	42.50 ± 1.04	< 0.01
AD	41.75 ± 1.00	< 0.01
GD	22.88 ± 1.26	
GD (before DCZ injection)	41.00 ± 2.75	< 0.01
Fecal mass (g)		
SS	2.02 ± 0.06	< 0.01
SD	2.03 ± 0.09	< 0.01
AD	1.92 ± 0.04	< 0.01
GD	0.81 ± 0.04	
GD (before DCZ injection)	1.95 ± 0.11	< 0.01
Fecal water content (%)		
SS	40.07 ± 0.86	< 0.01
SD	42.22 ± 0.78	< 0.01
AD	40.89 ± 0.38	< 0.01
GD	28.86 ± 2.18	
GD (before DCZ injection)	45.84 ± 1.17	< 0.01

Abbreviation: SEM, standard error of mean.

* Compared with the GD group.

In addition, the changes in GITT and colonic bead expulsion time are shown in Figure S1. We found that following DCZ injection, the GD group exhibited a longer GITT and colonic bead expulsion time compared with the SS (GITT: [110.49 ± 3.42] min vs. [80.31 ± 3.76] min; bead expulsion time: [10.34 ± 1.00] min vs. [5.52 ± 0.24] min), SD (GITT: [110.49 ± 3.42] min vs. [81.82 ± 7.73] min; bead expulsion time: [10.34 ± 1.00] min vs. [5.55 ± 0.30] min), and AD groups (GITT: [110.49 ± 3.42] min vs. [82.11 ± 7.57] min; bead expulsion time: [10.34 ± 1.00] min vs. [5.75 ± 0.31] min) (all *p* < 0.05). Notably, the GD group also exhibited a significantly longer GITT and colonic bead expulsion time after DCZ injection compared with those at baseline (GITT: [110.49 ± 3.42] min vs. [87.84 ± 6.90] min; bead expulsion time: [10.34 ± 1.00] min vs. [4.92 ± 0.28] min), before DCZ injection (GITT: [110.49 ± 3.42] min vs. [85.16 ± 7.21] min; bead expulsion time: [10.34 ± 1.00] min vs. [4.87 ± 0.39] min), and during the recovery period (GITT: [110.49 ± 3.42] min vs. [78.34 ± 7.57] min; bead expulsion time: [10.34 ± 1.00] min vs. [5.29 ± 0.32] min) (all *p* < 0.01).

### 3.2 Gi‐DREADD‐Treated Mice Exhibited Decreased Colonic Motility

**FIGURE 3:**
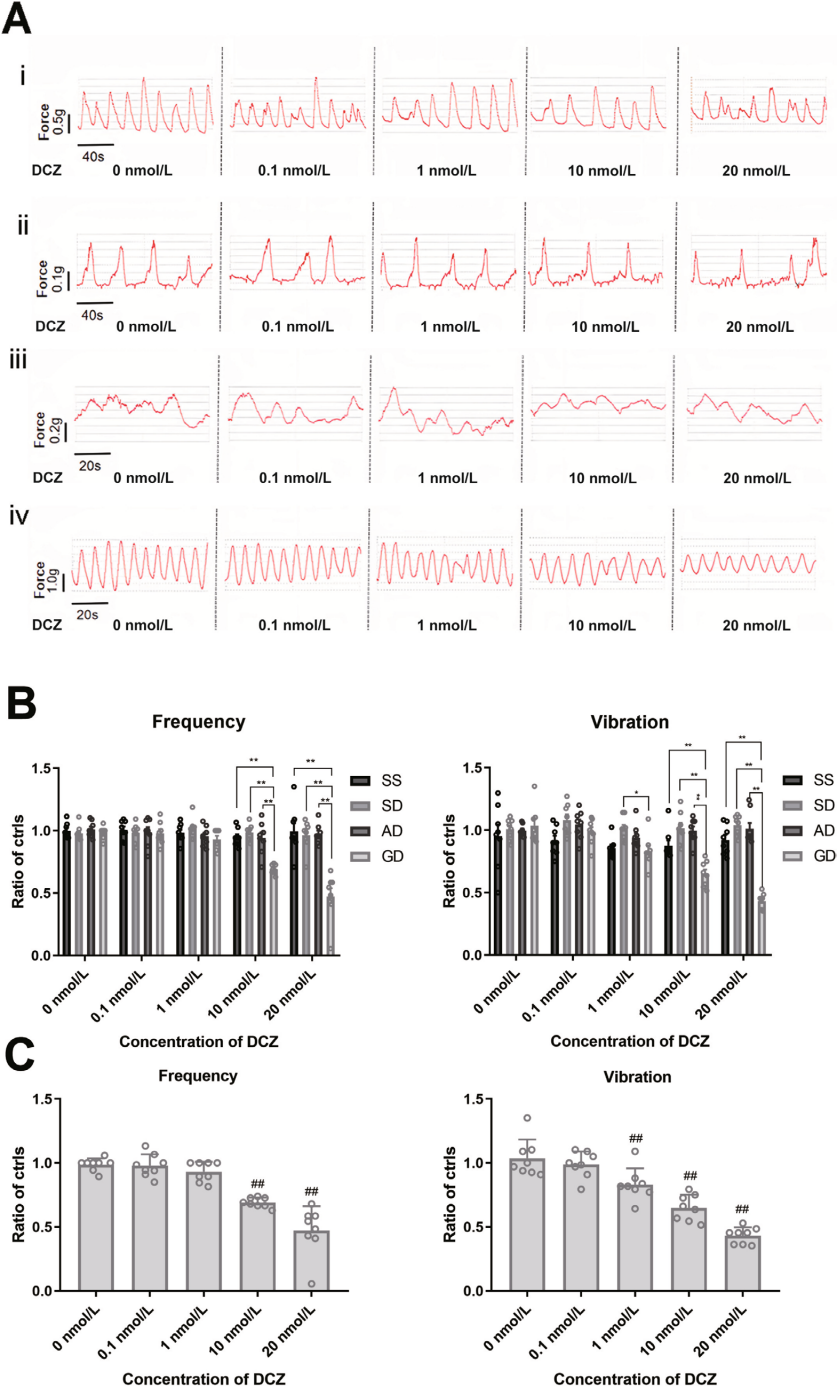
The isometric muscular force of colon tissues of the mice in the normal saline + normal saline (SS), normal saline + deschloroclozapine (DCZ) (SD), adeno‐associated virus (AAV)‐Cre + DCZ (AD), and Gi‐designer receptor exclusively activated by designer drug (Gi‐DREADD) + DCZ (GD) groups treated with DCZ of different concentrations. (A) Waves of the isometric muscular force of the colon under different concentrations of DCZ (i, SS; ii, SD; iii, AD; and iv, GD). Relative ratios to that of 0 nmol/L of the frequency and vibration of the isometric muscular force in (B) the four groups and (C) the GD group. All data are expressed as mean ± standard error of mean (*n* = 8 per group). *p < 0.05 and **p < 0.01 compared with GD group. ^##^ *p* < 0.01 compared with the 0 nmol/L DCZ subgroup in GD.

The isometric muscular force in different groups treated with different quantities of DCZ is shown in Figure 3A. With the DCZ concentrations being 10 and 20 nmol/L, the GD group had a lower frequency and amplitude of the waveform compared to those treated with other DCZ concentrations in the same group as well as to those in the SS, SD, and AD groups. The ratios (relative to that of 0 nmol/L DCZ) of the frequency and amplitude of colonic tissues in Gi‐DREADD‐treated mice decreased after the administration of 10 nmol/L DCZ, when compared with the SS (frequency: 0.69 ± 0.01 vs. 0.95 ± 0.02, *p* < 0.01; amplitude: 0.65 ± 0.04 vs. 0.88 ± 0.04, *p* < 0.01), SD (frequency: 0.69 ± 0.01 vs. 0.99 ± 0.02, *p* < 0.01; amplitude: 0.65 ± 0.04 vs. 1.02 ± 0.04, *p* < 0.01) and AD groups (frequency: 0.69 ± 0.01 vs. 0.94 ± 0.05, *p* < 0.01; amplitude: 0.65 ± 0.04 vs. 1.00 ± 0.03, *p* < 0.01). Similarly, with 20 nmol/L DCZ administration, the ratios of frequency and amplitude in Gi‐DREADD‐treated mice were lower than those of the SS (frequency: 0.47 ± 0.07 vs. 0.99 ± 0.07; amplitude, 0.43 ± 0.02 vs. 0.92 ± 0.04, *p* < 0.01), SD (frequency: 0.47 ± 0.07 vs. 0.96 ± 0.04, *p* < 0.01; amplitude: 0.43 ± 0.02 vs. 1.04 ± 0.03, *p* < 0.01), and AD groups (frequency: 0.47 ± 0.07 vs. 0.97 ± 0.03, *p* < 0.01; amplitude: 0.43 ± 0.02 vs. 1.01 ± 0.05, *p* < 0.01) (Figure 3B). While in the Gi‐DREADD‐treated mice, the ratios of the frequency and amplitude with DCZ at 10 nmol/L (frequency: 0.69 ± 0.01 vs. 0.99 ± 0.02, *p* < 0.01; amplitude: 0.65 ± 0.04 vs. 1.04 ± 0.05, *p* < 0.01) and 20 nmol/L (frequency: 0.47 ± 0.07 vs. 0.99 ± 0.02, *p* < 0.01; amplitude: 0.43 ± 0.02 vs. 1.04 ± 0.05, *p* < 0.01) were significantly lower than those treated with 0 nmol/L DCZ (Figure 3C).

### 3.3 The Virus Was Specifically Transduced Into the ENS Without Excitatory–Neural Favor

**FIGURE 4:**
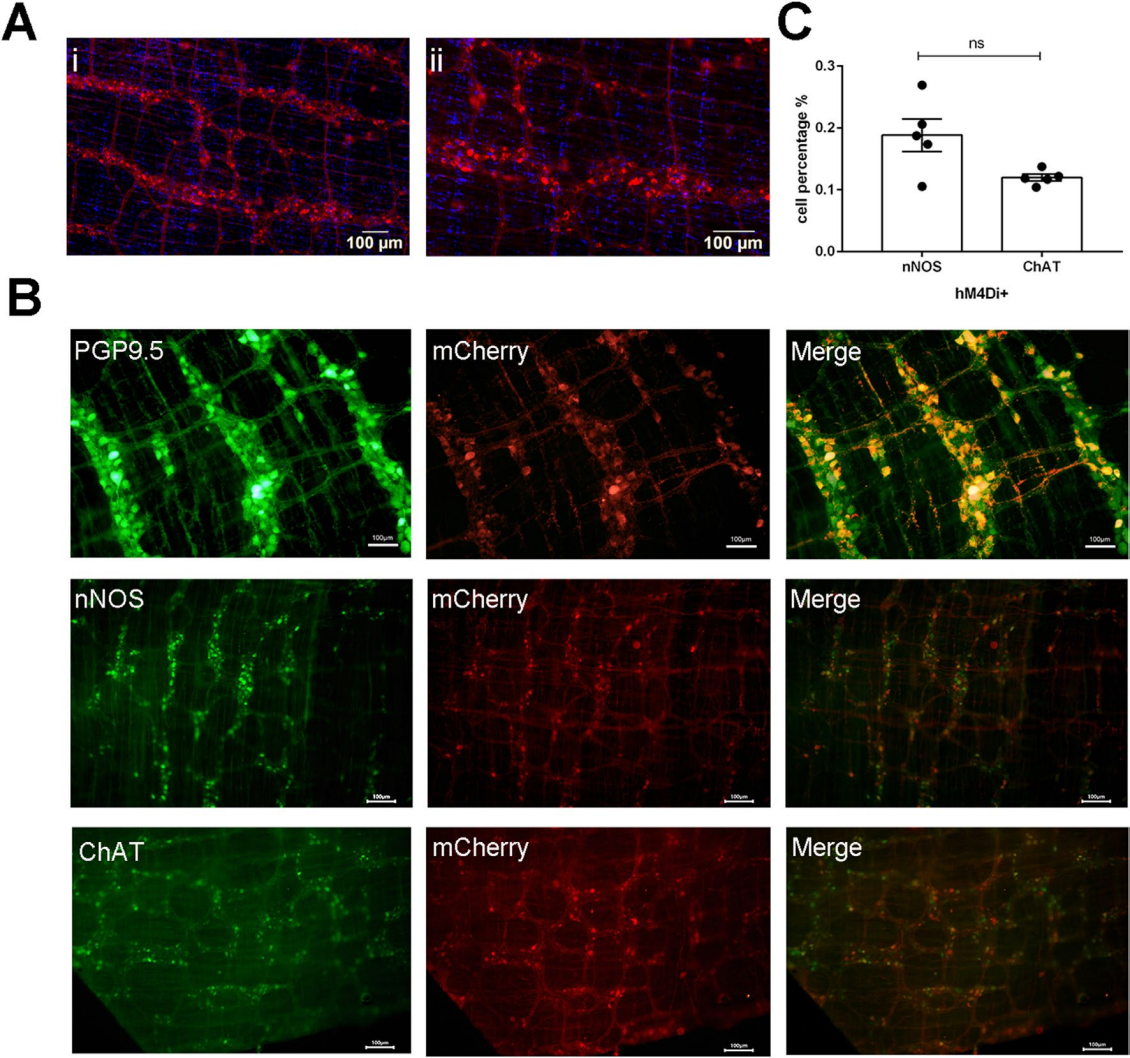
Immunohistochemistry of the enteric nervous system (ENS) in Gi‐designer receptor exclusively activated by designer drug (Gi‐DREADD)‐treated mice. (A) ENS of the Gi‐DREADD‐treated mice were stained for hM4Di‐positive virus (red) and the nucleus (blue). Magnification: i, ×10; ii, ×20. (B) PGP9.5, neuronal nitric oxide synthase (nNOS), choline acetyltransferase (ChAT), and mCherry co‐localization in the ENS. Upper panel, the transduction of neurons in the ENS was determined by examining the colocalization of green fluorescent neuron (PGP9.5) expression in hM4Di‐positive cells (colocalization in orange). Middle and lower panels, distribution and co‐localization of nNOS, ChAT with mCherry in the ENS, respectively. (C) Comparison of the percentages of the transduced neuron cells of nNOS and ChAT in hM4Di‐positive cells. (A,B) The scale bar represents 100 μm. Data are expressed as mean ± standard error of mean (*n* = 5 per group). ns, not significant.

The spatial and cellular expressions of hM4Di were examined by immunohistochemistry to verify the distribution of viral transfection. The mCherry‐labeled neurons representing the AAV9‐hM4Di virus were mainly observed throughout the ENS in Gi‐DREADD‐treated mice (Figure 4A). Immunostaining of mCherry in combination with PGP9.5 showed that the area of PGP9.5‐positive expression was highly coincident with that of mCherry, suggesting high efficiency of viral transfection to neurons by the injection of AAV9‐hM4Di virus into the intestinal wall. Immunohistochemical staining of nNOS and ChAT co‐localized with mCherry demonstrated that the regions expressing nNOS and ChAT were also highly consistent with those expressing mCherry (Figure 4B). Moreover, the double staining showed that the amount of co‐localization of nNOS and mCherry against all transduced neurons was not significantly different from that of ChAT and mCherry (Figure 4C).

### 3.4 Concentrations of Excitatory Neurotransmitters Decreased in Gi‐DREADD‐Treated Mice

**FIGURE 5:**
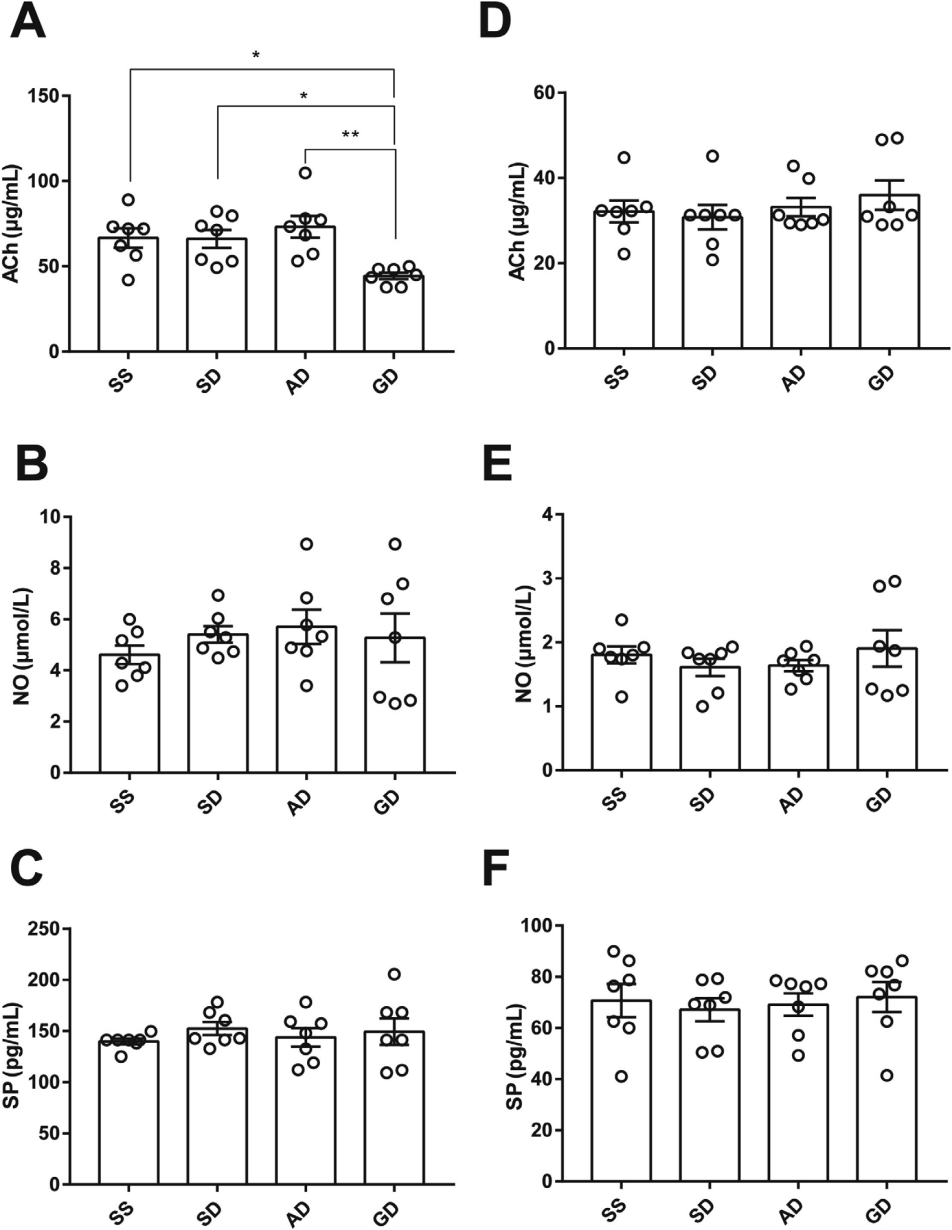
Levels of neurotransmitter receptors in (A–C) tissue supernatant and (D–F) serum of mice in the normal saline + normal saline (SS), normal saline + deschloroclozapine (DCZ) (SD), adeno‐associated virus (AAV)‐Cre + DCZ (AD), and Gi‐designer receptor exclusively activated by designer drug (Gi‐DREADD) + DCZ (GD) groups. A and D, acetylcholine (ACh); B and E, nitric oxide (NO); C and F, substance P (SP). All data are expressed as mean ± standard error of mean (*n* = 7 per group). *p < 0.05 and **p < 0.01 compared with the GD group.

In the colonic tissue supernatant, the level of excitatory neurotransmitter ACh in the Gi‐DREADD‐treated mice was lower than that of the mice in the SS ([44.41 ± 1.89] μg/mL vs. [66.71 ± 5.63] μg/mL, *p* = 0.023), SD ([44.41 ± 1.89] μg/mL vs. [66.18 ± 5.20] μg/mL, *p* = 0.027) and AD group ([44.41 ± 1.89] μg/mL vs. [73.21 ± 6.40] μg/mL, *p* < 0.01) (Figure 5A). While no difference was observed in the concentrations of NO and SP among groups (Figure 5B,C). However, when evaluating the serum levels of neurotransmitters, NO, SP, and ACh expressions did not show significant differences among groups (Figure 5D–F).

### 3.5 Ca^2+^ Transient of ENS in Gi‐DREADD‐Treated Mice Was Inhibited by DCZ

**FIGURE 6:**
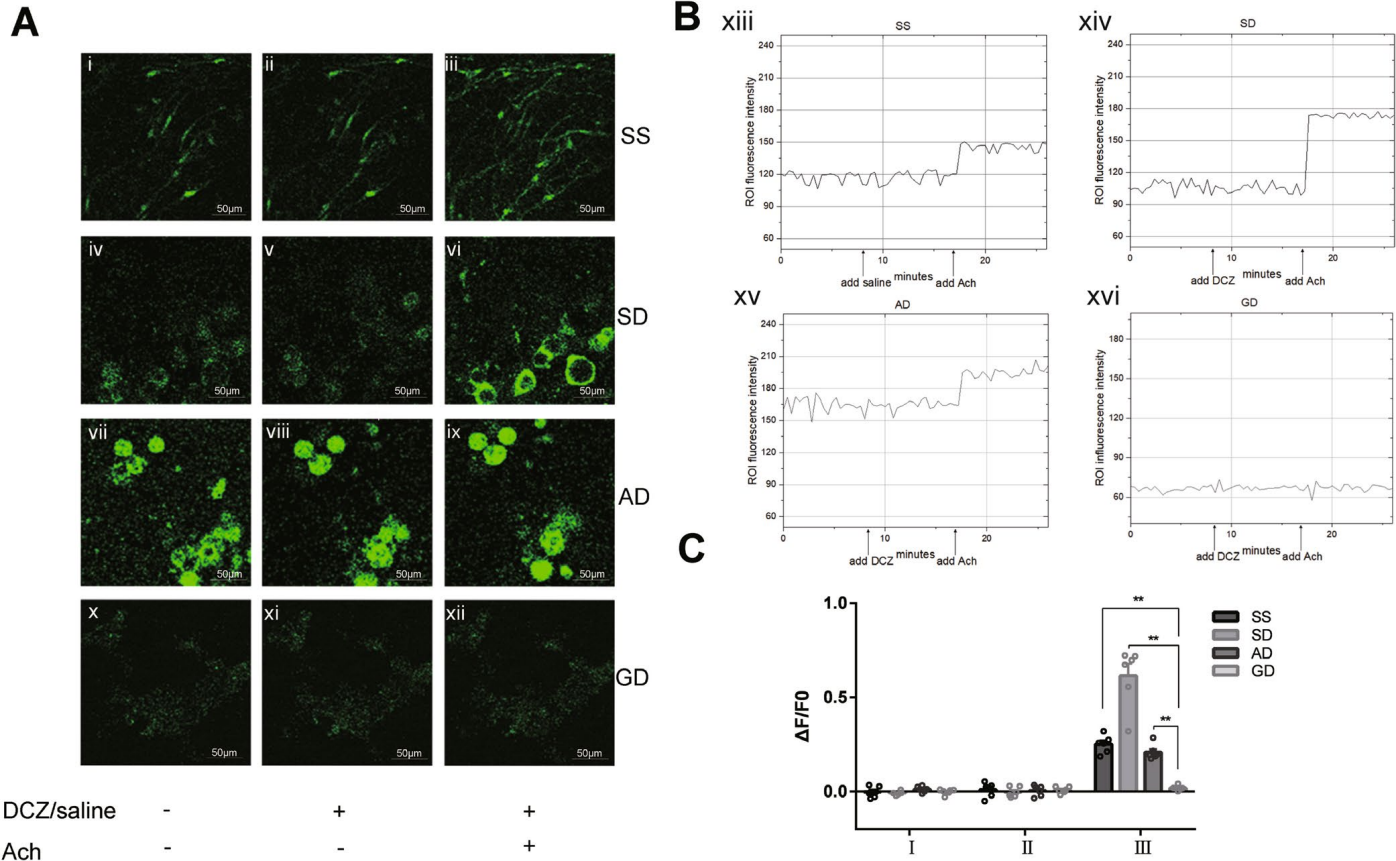
Calcium transient of isolated enteric nervous system (ENS) after adding 100 nmol/L acetylcholine (ACh) by deschloroclozapine (DCZ) (20 nmol/L) treatment. (A) Calcium ion transient of ENS in mice of the normal saline + normal saline (SS), normal saline + DCZ (SD), adeno‐associated virus (AAV)‐Cre + DCZ (AD), and Gi‐designer receptor exclusively activated by designer drug (Gi‐DREADD) + DCZ (GD) groups, as the fluorescent calcium indicator fluo‐4 (green) was monitored. i, iv, vii, x, enteric neurons in the medium; ii, enteric neurons of the SS group; v, viii, xi, enteric neurons of the SD, AD, and GD groups treated with DCZ; iii, vi, ix, xii, enteric neurons treated with ACh and DCZ. The scale bar represents 50 μm. (B) The region of interest (ROI) fluorescence intensity changes after adding DCZ (at the 9th min) and ACh (at the 17th min). xiii, SS group; xiv, SD group; xv, AD group; xvi, GD group. (C) Comparison of the fluorescence intensity change relative to the baseline value (ΔF/F0) of the calcium ion transient following ACh treatment under the previous DCZ treatment in the four groups (I, DCZ/normal saline [−] ACh [−]; II, DCZ/normal saline [+] ACh [−]; and III, DCZ/normal saline + ACh). All data are expressed as mean ± standard error of mean (*n* = 6 per group). **p < 0.01.

Calcium ion transient represents the activity of neurons to some extent. The activity of isolated enteric nerves in different groups of mice with DCZ treatment was observed by recording the fluorescence intensity (Figure 6A). Analysis of Ca^2+^ fluorescence in the ROI of the image revealed that in the SD, AD, and GD groups, the initial administration of DCZ did not induce any Ca^2+^ transient in enteric neurons. Normal saline did not change the Ca^2+^ fluorescence in the SS group as well. When ACh was added to the colonic tissues, the fluorescence intensity remained unchanged in Gi‐DREADD‐treated mice (Figure 6B xvi), while Ca^2+^ transient was evoked in the other three groups (Figure 6B xiii–xv). The specific value of ΔF/F0 in Gi‐DREADD‐treated mice after adding ACh was much lower than that of the other three groups (SS: 0.02 ± 0.01 vs. 0.25 ± 0.02, *p* < 0.01; SD: 0.02 ± 0.01 vs. 0.62 ± 0.06, *p* < 0.01; AD: 0.02 ± 0.01 vs. 0.21 ± 0.02, *p* < 0.01) (Figure 6C).

## 4 Discussion

To our knowledge, this is an innovative application of DREADDs in the engineering of modified receptors to mediate STC models within the ENS. AAV9‐hM4Di was successfully transduced with both CHAT‐ and nNOS‐positive enteric neurons. Lower contraction of circular colonic muscle and prolonged GITT in Gi‐DREADD‐treated mice resulted in decreased quantity of fecal pellets and harder stools due to decreased ACh in the colonic tissues. Also, Ca^2+^ transient induced by ACh in enteric neurons was not observed in the colonic tissues of Gi‐DREADD‐treated mice in vitro.

Systemic delivery of AAV has been shown to access non‐target organ tissues and neurons of the CNS [20, 21]. A lower level of transduction was displayed in the myenteric plexus of neonatal mice after intravenous administration of AAV9 [22]. Direct injection of the AAV9 vector into the descending colon of the mice can result in a high percentage of neuronal transduction within the ENS [23]. AAV9‐hM4Di injection in the current study achieved highly efficient transduction limited to neurons of the ENS 4 weeks after injection. Because disorders of the ENS localized to different areas along the GI tract are the main reason for GI motility disorders, providing a model system of site‐specific innervation to the ENS is necessary. Regulation of neural activity in the ENS with AAV9‐hM4Di vectors, using the method herein, may pave the way for more accurately recapitulating disorders of the ENS.

In line with previous studies wherein direct injection of AAV9 into the colon of mice [23], this study showed that the distribution of Gi‐DREADD transfection was on the myenteric plexus, as almost all virus‐transfected cells were positive for PGP9.5. Activation of the myenteric plexus orchestrates the SIP syncytium to generate propagating contractions, thereby facilitating distal propulsion of luminal contents. In the SIP syncytium, ICC generates Ca^2+^‐activated inward currents according to signals transmitted by nerve cells, thus producing depolarization and excitation effects in the SIP syncytium, while PDGFRα^+^ cells generate opposite effects. ICC and PDGFRα^+^ cells are electrically coupled with SMCs and coordinate muscle activity [24]. In our experiment, inhibitory Gi‐GPCRs were activated via DCZ, the myenteric plexus input weakened impulse, and the ionic activity in ICC and PDGFRα^+^ cells was both decreased. With weakened current transmitting to SMCs, intestinal contraction was then attenuated. The abovementioned process resulted in impaired colonic motility and decreased fecal quantity in Gi‐DREADD‐treated mice. The transfection included both ChAT and nNOS, which are both important components of intestinal motor nerves. There appeared to be no significant difference in the percentage of neurons positive for nNOS and ChAT, indicating that there was no specific bias in virus transfection on neurons and that the activity of both neurons was suppressed. Notably, although nNOS neurons are inhibitory neurons, ChAT neurons are not only excitatory neurons; some of them can induce inhibitory neural activity as well, while the neurotransmitter ACh only directs the excitatory action of ChAT neurons [25]. Consistent with our results, the neurotransmitter ACh was found to decrease in the colonic tissues of STC patients [26] and loperamide‐induced mouse model [27]. However, the inhibitory neurotransmitter NO did not increase in Gi‐DREADD‐treated mice in our study, which differed from that of the STC patients [28]. This might be due to the reason that Gi‐DREADDs inhibit, rather than stimulate, the release of neurotransmitters, thus the ACh level decreased, but that of NO remained unchanged. Our study showed that, with the activation of Gi‐GPCRs, the release of ACh from the synaptic terminals was reduced, weakening the excitatory effect of ChAT neurons in the ENS, which further inhibited intestinal peristalsis, resulting in STC in Gi‐DREADD‐treated mice.

So far, the exact pathophysiology of STC remains to be investigated. Decreased intestinal nerve activity, accompanied by changes in neurotransmitters and muscle activity, may be observed in most STC patients [29, 30]. In our study, GPCRs on the surface of neuron cells are transformed into Gi‐GPCRs with viruses successfully transduced to nerves. The hM4D receptor is designed by mutation Y3.33 and A5.46, transferring to DCZ‐activated Gi‐DREADDs. When Gi‐DREADDs are activated, ACh release in neuronal terminals is reduced, and the excitability of receptor‐operated channels on the nerve membrane surface is weakened. Therefore, we speculate that extracellular infiltration through Ca^2+^‐permeable channels and intracellular release from the endoplasmic reticulum is inhibited, thus inhibiting the activity of adenylate cyclase (AC) in cells, and decreasing the level of second messenger cyclic adenosine monophosphate (cAMP). Protein phosphorylation in neurons is then reduced as the result of a decreased activation of protein kinase A (PKA). Meanwhile, a robust hyperpolarization of the membranes can be observed as G protein inward‐rectifying potassium channels (GIRKs) in neural cells are activated, silencing the neuronal firing [31, 32]. The levels of these signaling molecules can affect Ca^2+^ levels by influencing Ca^2+^ channels in feedback [33, 34]. More convincingly, the present study showed that the Ca^2+^ transient of ENS from the Gi‐DREADD‐treated mice was significantly reduced after DCZ treatment. The decreased concentration of cytoplasm Ca^2+^ then reduced the levels of excitatory neurotransmitters. Thus, ENS activity was reduced, and intestinal motility was impaired.

Loperamide is one of the most commonly used drugs to establish the STC model. It can reduce neuronal activity and inhibit muscle contraction via the activation of opioid receptors [35, 36]; however, it has mainly been used to study the effects of laxatives on constipation [37]. However, due to the presence of off‐target effects after systemically delivered agents, a more precise and effective way to modulate the ENS function is required. We found that Gi‐DREADD transfection resulted in reduced and hard fecal pellets, especially after DCZ administration. Indeed, these results strongly demonstrated that the inhibitory effects of colonic motility were mediated via specifically activating the reformed Gi‐GPCRs. In addition, during the validity period of viral transfection, injection of DCZ may be applied at any time to induce STC, without continuous drug accumulation. Also, the regulation of ENS is reversible. Our study showed that the quantity, mass, and water content of the fecal samples decreased only when Gi‐GPCRs were activated by DCZ. Once the activation stops, defecation of the mice recovered to normal level. Another innovative technology, optogenetics, has been applied to induce the electrical activity of enteric neurons [38]. Viral vectors and transgenic animals are most commonly used to target the improved optogenetic actuators in ENS. Muscle contraction is observed under optical illumination [39]. Notably, the light activating the indicators must be with the correct wavelength for sufficient stimulation, and single‐ or two‐photon stimulation may cause severe injury [40, 41]. Therefore, our method for model establishment may be safer and simpler. DREADDs enable non‐invasive regulation, as the receptors can be activated by simple intraperitoneal injection. In addition, DREADD‐based approaches eliminate the need for complex optical equipment and continuous photo stimulation.

There were some limitations to this study. Temporal control is not emphasized in DREADDs, compared with optogenetics [42]. DREADDs can be maintained for a long time period, especially when applied in vivo; therefore, the remaining effective time could not be evaluated. The number of mice used in our experiment was relatively small; our results should be further verified in further studies including more animals.

In conclusion, we provided novel insights that applying the Gi‐DREADDs can evoke the development of STC in mice, which is related to the inhibition of the enteric neurons via inhibited Ca^2+^ transient and decreased release of ACh in neuron cells of the ENS. This finding offers an available STC model and provides the potential of chemogenetic technologies for considerable clinical therapeutics.

## Conflicts of Interest

The authors declare no conflicts of interest.

## Supporting information


**Figure S1.** Comparison of (A) gastrointestinal transit time (GITT) and (B) bead expulsion time among the saline + saline (SS), saline + deschloroclozapine (DCZ) (SD), adeno‐associated virus (AAV)‐Cre + DCZ (AD), and Gi‐designer receptor exclusively activated by designer drug (Gi‐DREADD) + DCZ (GD) groups. All data are expressed as mean ± standard error of mean (*n* = 6 per group). *p < 0.05, **p < 0.01, and ***p < 0.001 compared with the GD group after DCZ injection.
