# Supplementary figures and images for: DREADDs‐Based Chemogenetics Induced Slow Transit Constipation via Inhibition of Enteric Neurons
Source: J Dig Dis. 2025 Apr 14;26(1-2):62–73. doi: 10.1111/1751-2980.13344 (PMC12038534; doi:10.1111/1751-2980.13344)

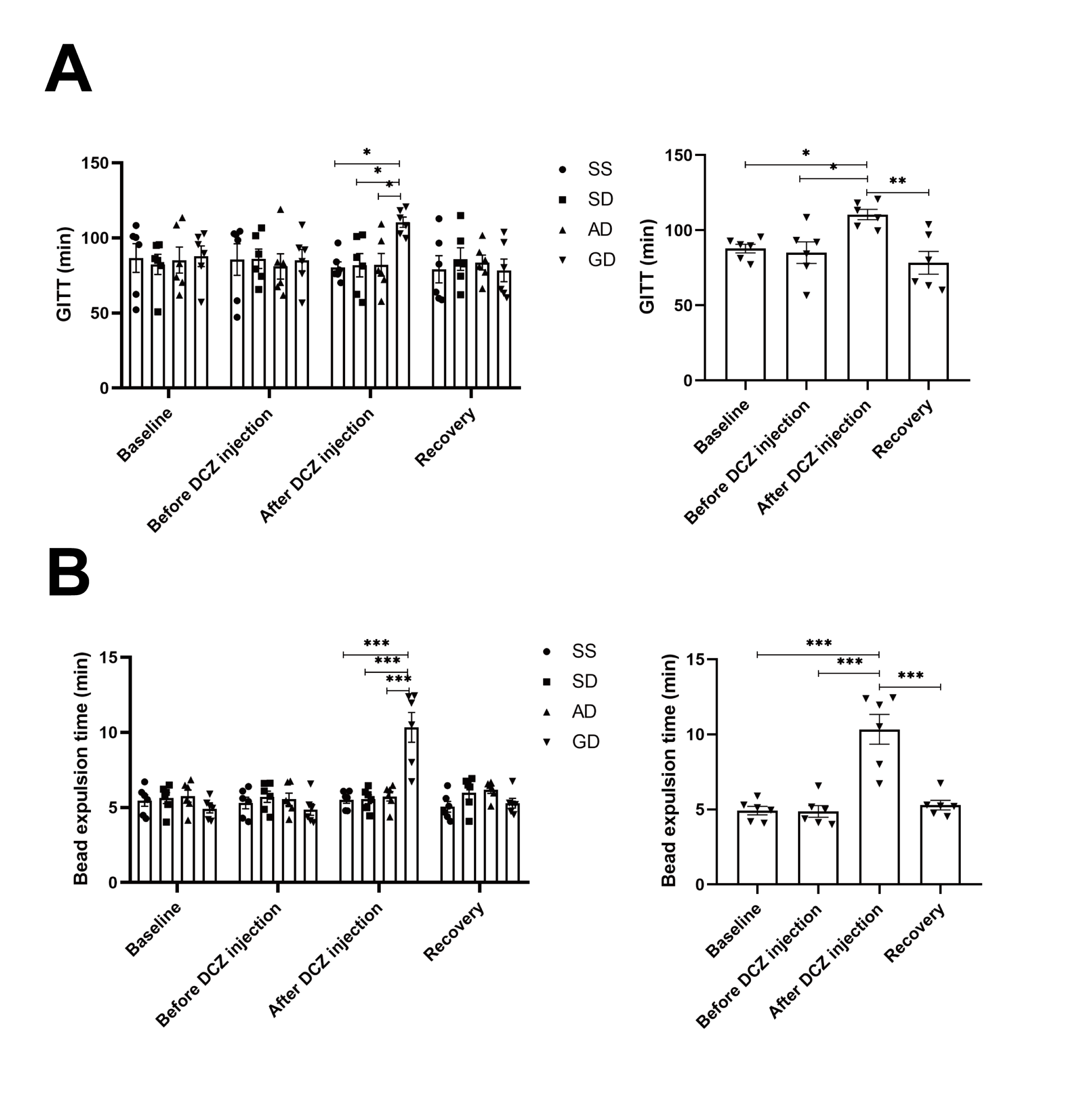

Supplement: Supplementary file 1 — Figure S1. Comparison of (A) gastrointestinal transit time (GITT) and (B) bead expulsion time among the saline + saline (SS), saline + deschloroclozapine (DCZ) (SD), adeno‐associated virus (AAV)‐Cre + DCZ (AD), and Gi‐designer receptor exclusively activated by designer drug (Gi‐DREADD) + DCZ (GD) groups. All data are expressed as mean ± standard error of mean (n = 6 per group). *p < 0.05, **p < 0.01, and ***p < 0.001 compared with the GD group after DCZ injection. [file CDD-26-62-s001.tif]
